# Pain and symptoms of depression: international comparative study on selected factors affecting the quality of life of elderly people residing in institutions in Europe

**DOI:** 10.1186/s12877-019-1164-5

**Published:** 2019-05-27

**Authors:** Izabela Wróblewska, Dorota Talarska, Zuzanna Wróblewska, Robert Susło, Jarosław Drobnik

**Affiliations:** 1 Gerontology Unit, Public Health Department, Faculty of Health Sciences, Wrocław Medical University, Poland 5 Bartel St, 50-618 Wrocław, Poland; 2 Department of Preventive Medicine, Faculty of Medical Sciences, Poznań University of Health Sciences, Poland 6 Święcicki St, 60-781 Poznań, Poland; 3 Karkonosze College in Jelenia Góra KPSW, 18 Lwówiecka St, 58-503 Jelenia Góra, Poland

**Keywords:** Gerontology, Quality of life, Depression, Pain, Institutional care

## Abstract

**Background:**

As the number of elderly people is on the rise in societies throughout the world, providing them with optimal care is becoming a major demand, especially in the context of rising interest in institutional care. Quality of life is multidimensional notion and its perception depends highly on pain and mood levels. The aim of this study was to perform a comparative analysis of pain and depression symptoms in elderly people living in nursing homes in France, Germany, and Poland.

**Methods:**

The research carried out in years 2014–2016 involved female residents of nursing homes in France, Germany, and Poland: 190 women from each country, aged over 65 years and not previously diagnosed with advanced dementia, were included. Collection of medical, demographic, and anthropomorphic data from medical documentation was followed by interviews with each senior and her caregiver. A questionnaire of authors’ own devising was used, along with the Beck Depression Inventory (BDI) and the scale of Behavioral Pain Assessment in the Elderly (DOLOPLUS). The results were subjected to statistical analysis, *p* < 0.05 was accepted as threshold of statistical significance.

**Results:**

The main health complaints of nursing homes’ residents were constipation, diarrhea, back pain and dizziness. 44,38% of the residents self-assessed their health status as bad and complained of suffering pain (83,33%) and sleeping problems (72,98%) within the last month. According to BDI the average score was 17.01 points and 44,38% of seniors were free from depression or depressed mood. The average DOLOPLUS result was 8.86 points.

**Conclusion:**

There are no significant differences, neither in prevalence of pain and symptoms of depression nor in average levels of quality of life, in elderly residents in institutions in the three studied European countries. The decrease in quality of life is mainly due to various complaints and pain and there is a close relationship between health status and quality of life. Further research should be performed in order to study interdependencies between the occurrence of pain and depression, including primary reasons leading to both phenomena. The recognition of factors that induce pain complaints and mood depression in elderly people will contribute to improving their comfort.

## Background

As the number of elderly people is on the rise in societies throughout the world, providing the elderly with optimal care is becoming a major aim of social and medical service activities [1, 2]. This issue becomes even more crucial with the increase in interest in institutional care [3]. A high standard of service means meeting all the biological, psychological, and social needs of seniors to the greatest degree possible, and in particular involves ensuring a good quality of life [4]. Quality of life is a multidimensional notion, an element of some operationalized health definitions, and an indispensable component of human health status assessment [5]. It remains closely associated with health status (health-related quality of life) [6], as disease is often associated with pain and suffering, causes feelings of danger and isolation, and lowers well-being generally, ultimately often triggering the onset of depression. Comorbidities and their symptoms, including headache, dizziness, syncope, chest pain, heart palpitations, abdominal ache, back and joint pain, feelings of heaviness in the legs, dyspnea, nausea, vomiting, flatulence, constipation and diarrhea all add a significant burden to elderly patients’ suffering. Health status can be objectively assessed using biomedical indicators; some aspects, however, are difficult to characterize with such parameters: these include a lack of pain and other physical or psychical complaints [7].

Analysis of factors that are detrimental to quality of life can help to find and deliver forms of care that capable of limiting or even eliminating them completely. [8, 9]. A broader look at the elderly’s needs necessitates both the introduction of humanistic-oriented care and the implementation of a truly holistic health concept. This would include studying the subjective perception of quality of life, including its crucial limiting elements, such as pain and depressed mood [10]. Consequently, the aim of this study was to perform a comparative analysis of pain and depression symptoms as selected important factors affecting the quality of life of elderly people living in nursing homes in France, Germany, and Poland.

## Methods

### Participants

The research took place in the years 2014–2016 and involved 570 residents of nursing homes in France, Germany, and Poland. The inclusion criteria were age over 65 years and lack of diagnosed advanced dementia, defined as a score of 0–10 points in the Mini Mental State Examination (MMSE), which is a well accepted tool commonly used in the routine evaluation of elderly patients of nursing homes. Polish nursing homes segregate sexes, and only those with female patients agreed to be involved into the study; however, in French and German institutions participating in the study, male patients constituted only a small percentage of the residents. Thus, for the sake of data comparability, male residents were not enrolled in the study. As a result, the analysis included 190 women from each country.

### Data collection

After written informed consent to participate in the study had been collected from patients, the relevant medical, demographic, and anthropomorphic data were retrieved on the basis of the available medical documentation. These included age, sex, place of residence, marital status, educational level, living conditions before moving into the nursing home, length of stay in the nursing home, use of antidepressant and anxiolytic drugs, and types and profiles of current illnesses and complaints—especially the most common comorbidities and their most burdensome symptoms. The Beck Depression Inventory (BDI) and the scale of Behavioral Pain Assessment in the Elderly (DOLOPLUS) were used as diagnostic tools; these are well accepted by patients and have been evaluated by the scientific community. The BDI scale involves 21 questions regarding various depression symptoms, with three possible answers each, mirroring the increasing intensity of symptoms scored from 1 to 3 points; a result of 0–11 points means no depression, 12–26 points means mild depression, 27–49 points refers to mid-intense depression, and 50–63 points means severe depression. DOLOPLUS is tool for detecting pain-related behavioral alterations: this scale consists of three parts measuring somatic reactions, psychomotor skills and psychosocial competences; scoring 5 or more points out of the 30 available is interpreted as confirming pain-related suffering and is considered justification for introducing analgesic treatment. We also carried out an interview with each senior and her caregiver, and gathered empirical material with the use of a questionnaire of our own devising, which included questions concerning subjective rating of health status, quality of life, quality of sleep, and intensity of pain, using a scale ranging from 0 to 10 points (the worst and the best rating, respectively).

### Data analysis

The results concerning women residing in Polish, French and German nursing homes underwent statistical analysis including three respective groups; the values of the parameters measured on the nominal scale were expressed as absolute values (N) and percentages (%). The impact of the independent variables on the subjective assessment of the quality of life was expressed in points (for the BDI and DOLOPLUS scales), and was analyzed using multiple regression. The quality of life model included analyses of multiple factors, among them: X_1_ (age), X_2_ (place of residence), X_3_ (marital status), X_4_ (living conditions), X_5_ (educational level), X_6_ (independence in performing basic activities), X_7_ (dependence from other person’s care). General model of multiple and linear regression was used:

$$ {\displaystyle \begin{array}{c}Y=f\left(X,\beta \right)+\varepsilon \\ {}Y=f\left(X+{\varepsilon}_x{,}_{,}\beta \right)+\varepsilon \\ {}Y={\beta}_0+{x}_1{\beta}_1+{x}_2{\beta}_2+\dots +{x}_n{\beta}_n+\varepsilon \end{array}} $$

where: *X* means vector of independent variables; *Y* means dependent variable; *β* means model coefficient; *f*(*X*, *β*) means regression function of real numbers values; *F* means statistics F value in regression; *df* means degree of freedom; *p* means statistical significance; *R*^*2*^ means coefficient of determination; *E* means random error; the least squares method algorithm was applied. The accepted models of analysis can be considered trustworthy because for the BDI scale data *R*^*2*^ = 0,7308 and for the DOLOPLUS scale data *R*^*2*^ = 0,7449; and for both datasets the variance analysis points at the error value close to the null (Tables 1 and 2). We took statistically significant data to have a level of *p* < 0.05. We also calculated the arithmetical mean, standard deviation (SD), median, and the minimum and maximum values for all parameters. Statistical analysis was performed using Microsoft Excel spreadsheet and Statistica 8.0 software.

**Table 1:** Variance analysis for the variable: results of Beck Depression Inventory (BDI) scale examination of the elderly living in nursing homes

	Sum of squares	df	Average of squares	F	*p*
Regression	32,624,12	99	329,5366	11,39,608	0,00
Remainder	8096,66	280	28,9167		
Total	40,720,79

**Table 2:** Variance analysis for the variable: results of DOLOPLUS scale examination of the elderly living in nursing homes

	Sum of squares	df	Average of squares	F	*p*
Regression	10,202,13	99	103,0518	12,17,637	0,00
Remainder	2369,71	280	84,633		
Total	12,571,84				

## Results

The mean age of the female residents of the Polish nursing homes was lower than that in the French and German homes. The majority were aged 80–89 years in Poland, and 90–99 years in Germany and France. At all the centers, the residents came mainly from large cities or towns and were single. In Poland, they had usually experienced good living conditions; those in France and Germany had experienced very good living conditions. Most of the Polish respondents had lived in a nursing home for 1–3 years, while in Germany and France the period was 4–9 years (Table 3).

**Table 3:** Group characteristics

	Total		Poland		France		Germany		Test result *p* < 0.05
	mean values	percentage	mean values	percentage	mean values	percentage	mean values	percentage	
Number of patients	570	100	190	33.33	190	33.33	190	33.33	
Age [years]:									
a) mean	85.67		79.29		88.87		88.87		
b) standard deviation	8.87		8.24		7.19		7.19		0.000
c) median	87		80.5		89		91		
d) minimum	65		65		70		71		
e) maximum	104		100		104		101		
Place of living^a^:									
a) village	109	19.12	35	18.42	40	21.05	34	17.89	0.007
b) city	461	81.86	155	81.58	150	78.95	156	82.09	
Marital status:									
a) single	176	30.87	55	28.95	60	31.58	61	32.00	0.054
b) married	60	10.52	23	12.11	16	8.42	21	11.05	
c) widow	334	58.59	112	58.95	114	60.00	108	56.84	
Staying in the nursing home:									
a) < 12 months	145	25.43	63	33.16	40	21.05	42	22.10	
b) 1–9 years	360	63.15	102	53.68	126	63.31	132	69.46	0.000
c) ≥10 years	65	11.39	25	13.15	24	12.63	16	8.41	

In total, physicians diagnosed 34 disease entities in all the women. The most frequent diseases were heart failure, hypertension, and dementia; least frequent were venereal diseases, psoriasis, and abdominal aorta aneurysm. Table 4 presents the disease entities that were most frequently diagnosed by physicians in the seniors. In contrast, among the 570 seniors, the most commonly reported complaints were constipation and diarrhea (*N* = 412); back pain (*N* = 339), dizziness (*N* = 224), concentration difficulties (*N* = 252), becoming easily fatigued (*N* = 196), and a feeling of sadness and mood depression (*N* = 171) were significantly less common.

**Table 4:** Disease entities

	Total		Poland		France		Germany		Test result *p* < 0.05
	mean values	percentage	mean values	percentage	mean values	percentage	mean values	percentage	
Number of patients	570	100	190	33.33	190	33.33	190	33.33	
Heart failure	554	97.19	196	103.16	171	90.00	187	98.39	0.469
Hypertension	317	55.61	103	54.21	108	56.84	106	55.78	0.741
Dementia	313	54.85	95	50.00	113	59.47	105	55.25	0.002^a^
Depression	276	48.50	109	57.37	80	42.11	87	45.78	0.701
Osteoporosis	241	42.45	79	41.58	82	43.16	81	42.63	0.495
General atherosclerosis	186	32.63	96	50.53	28	14.74	62	32.63	0.000^a^
Neoplastic disease	145	25.53	28	14.73	69	36.32	48	25.26	0.000^a^
Diabetes	123	21.52	49	25.79	33	21.58	41	21.57	0.077
Degenerative joint disease	120	21.05	64	33.68	16	8.42	40	21.05	0.000^a^
Thyroid gland diseases	76	13.42	16	8.42	35	18.42	25	13.15	0.004^a^
Condition after femoral bone fracture	64	11.22	26	13.68	17	8.95	21	11.05	0.145
Multiple sclerosis	52	9.12	18	9.47	17	8.95	17	8.95	0.859
Neurosis	48	8.42	26	13.68	6	3.16	16	8.42	0.000^a^
Condition after cholecystectomy	42	7.37	14	7.37	14	7.37	14	7.37	1.000
Myocardial infarction	36	6.32	10	5.26	14	7.37	12	6.31	0.400
Hydrocephalus	35	6.14	14	7.37	9	4.74	12	6.31	0.283
Parkinson’s disease	33	5.79	12	6.32	10	5.26	11	5.78	0.661

^a^Statistically significant values

Most of the nursing home residents self-assessed their health status as bad (*N* = 253); this applied to 48.42% of respondents in France, 44.73% in Germany, and 40.00% in Poland. As many as 317 respondents described their health status as good, very good, or excellent (20.00% of the Polish, 18.42% of the German, and 17.19% of the French seniors). The majority of the study population self-assessed their health as being somewhat worse or much worse than in the previous year (*N* = 302; 52.98%). The average subjective assessment of health in all the countries was 4.64 points. The lowest ratings were observed in France, and the highest in Poland.

Regarding physical pain, a large group of respondents reported having felt some pain within the last month (*N* = 475; 83.33%). This response was noted in 163 (28.59%) French, 158 (27.71%) Polish, and 154 (27.01%) German seniors. Most respondents complained of mild pain (204; 35.78%), followed by medium (152; 26.66%), strong (64; 11.22%), and very strong (41; 7.19%) pain. A majority of the Polish women complained of medium, strong, or very strong pain, while French women most frequently experienced mild pain. The majority of German women did not report any pain, and the largest group experiencing very strong pain was Polish.

We employed a subjective sleep scale ranging from 0 points, denoting bad sleep, to 10 points, referring to very good sleep. Most women in the group stated that they had trouble sleeping (*N* = 416; 72.98%); in the case of 196 (34.38%) seniors, this constituted a serious problem. The least frequent complaints about sleep disturbances were noted among Poles (77; 40.53%), with the most frequent among the German women (165; 86.84%). The average subjective assessment of the sleep of all the seniors was 6.85 points. Tables 5 and 6 present the 26 most frequent problems and complaints reported by the women, as well as the DOLOPLUS and BDI scores and the results of the questionnaire on patient health status.

**Table 5:** Problems and complaints

	Total		Poland		France		Germany		Test result *p* < 0.05
	mean values	percentage	mean values	percentage	mean values	percentage	mean values	percentage	
Number of patients	570	100	190	33.33	190	33.33	190	33.33	
Constipation, diarrhoea	412	72.28	132	69.47	139	73.16	141	74.21	0.323
Back pain	339	59.47	118	62.11	102	53.68	119	62.63	0.096
Dizziness	325	57.01	122	64.21	102	53.68	101	53.15	0.037^a^
Feeling of heavy legs	307	53.85	107	56.32	103	54.21	97	51.05	0.680
Arthralgia	291	51.05	106	55.79	83	43.68	102	53.68	0.018^a^
Feeling of sadness and mood depression	171	30.00	84	44.21	30	15.79	57	30.01	0.000^a^
Sleep disturbances	196	34.38	56	29.47	48	25.26	92	48.42	0.264
Continuous feeling of tiredness	159	27.89	69	36.32	37	19.47	53	27.90	0.014^a^
Concentration difficulties	252	44.21	88	46.32	80	42.11	84	44.22	0.293
Slowness/agitation	209	36.66	82	43.16	48	25.26	79	41.57	0.445
Appetite disturbances	169	29.64	22	11.58	91	47.89	56	29.47	0.000^a^
Degree of difficulty in everyday functioning									
a) no difficulty	27	4.73	18	9.47	0	0	9	4.73	
b) medium	338	59.29	92	48.42	133	70.00	113	59.47	0.398
c) high	205	35.96	80	42.11	57	30.00	68	35.78	
General subjective health assessment:									
a) good	151	26.48	48	25.26	53	27.89	50	26.31	
b) medium	166	29.12	66	34.74	45	23.68	55	28.94	0.462
c) bad	253	44.38	76	40.00	92	48.42	85	44.73	
Subjective physical pain:									
a) no pain	95	16.66	32	16.84	27	14.21	36	18.94	
b) medium	336	62.98	107	56.32	122	64.22	135	71.04	0.007^a^
c) strong	111	19.46	51	26.84	41	21.58	19	9.99	

^a^Statistically significant values

**Table 6:** Standard questionnaire results

Scales	Total	France	Poland	Germany	Test result *p* < 0.05
Health status:					
a) mean	4.64	4.58	4.69	4.65	
b) standard deviation	2.79	2.61	2.97	2.81	
c) median	5	5	6	5	0.713
d) minimum	0	0	0	0	
e) maximum	10	8	10	9	
Subjective sleep quality assessment:					
a) mean	6.84	6.97	6.73	6.82	
b) standard deviation	2.75	1.98	3.34	2.93	
c) median	8	7	8	8	0.401
d) minimum	0	2	0	1	
e) maximum	10	10	10	10	
Beck Depression Inventory (BDI):					
a) mean	17.98	17.01	18.94	17.99	
b) standard deviation	10.37	9.46	11.14	10.51	0.069
c) median	14.50	13	17	16	
d) minimum	1	3	1	2	
e) maximum	42	36	42	39	
Behavioural Pain Assessment in the Elderly (DOLOPLUS):					
a) mean	8.86	7.09	10.60	8.91	
b) standard deviation	5.76	2.96	7.18	7.14	0.000^a^
c) median	7.50	6	10	9	
d) minimum	0	2	0	1	
e) maximum	25	17	25	22	

^a^A statistically significant value

The results regarding symptoms of depression are comparable in all groups. The majority of the respondents did not present depression symptoms or depressed mood, according to the BDI scale (72; 37.89% for Poland; 97; 51.05% for France; 84; 44.21% for Germany); some patients had features of mild depression (52; 27.37% for Poland; 46; 24.21% for France; 49; 25.78% for Germany) or symptoms of medium or severe depression (respectively, 49; 25.79% and 17; 8.95% for Poland; 36; 18.95% and 11; 5.79% for France; 38; 20.00% and 19; 10.00% for Germany). The average BDI score was 17.01 points. We observed the lowest rates in France (17.01 points) and the highest in Poland (18.94 points).

These results are similar to those of the DOLOPLUS instrument (10.6% for Poland; 7.09% for France; 8.91% for Germany), which demonstrate the presence of various types of pain. The average DOLOPLUS result for the entire study population was 8.86 points; the lowest results were achieved in France (7.09 points), and the highest in Poland (10.60 points) (Table 6).

## Discussion

The major limitation of the study was only female elderly residents of nursing homes involvement as it does not allow to extrapolate the results directly onto the whole population of elderly people residing in nursing homes in the countries involved into the study; being aware of that the authors plan to carry out a complementary study involving the male elderly residents in the future. The other significant but unavoidable limitation - resulting from the characteristics of the diagnostic tools used which demanded at least a basic level of cooperation from the study participants - was exclusion from the study of the patients diagnosed with advanced dementia while such patients are almost always present among residents of nursing homes, although their shares differ heavily in different institutions so it is difficult to assess the actual importance of that limitation.

Our results show that the Polish female nursing home residents were on average younger than the French and German patients. The difference of over 9 years between their mean ages may result from higher living standards in Western Europe, along with higher disease detection rate there, and in particular the longer overall experience in the field of organized care of elderly people [11, 12]. As women in nursing homes for seniors are mostly single, their disabilities and other circumstances do not allow them to function independently and alone in their environment, even after the available necessary adjustments have been made. However, studies have shown that this may also be affected by differences in the conditions of the women’s original background and the resulting varied expectations of care [13, 14].

Consistently with the data provided in literature [15], the study results confirmed that multimorbidity among the residents of nursing homes is the rule: in each respondent, at least three disease entities typical of old age were present, of which the most common were hypertension, generalized atherosclerosis, heart disease, osteoporosis, and dementia. According to the literature, the syndromes of dementia are among the most frequent causes of the loss of independent life in the elderly; they cause a significant decrease in the quality of their life [16] as well as of that of their caregivers [17]; the results of this research also support this observation.

The demonstrated higher prevalence of cancer in the group also significantly impaired the patients’ functioning and worsened their general feeling. The fact that many of the symptoms in the seniors are also seen in various stages of cancer and other chronic diseases is an additional diagnostic problem [18]. Comorbidity, polypharmacy, and difficult-to-predict adverse events can cause pain, which often leads to feelings of physical discomfort—although this may also have a psychological background, and thus not respond to pharmacotherapy, but might be curable by psychotherapy [19].

The patients in the group studied here reported numerous everyday problems that affected their general feeling. Among the most important problems was constipation, leading to discomfort (observed in three quarters of the group), pain, and worsening of overall health status. Constipation is often signaled in advance by headache, abdominal distension, and flatulence. Constipation may be caused by movement limitations, difficulties in reaching the toilet, inappropriate diet, medication, lowered liquid intake, and slower peristalsis, all of which were observed in our patients [20].

The seniors also complained of concentration difficulties, tinnitus, and memory problems. These may be symptoms of physiological aging or of specific diseases [21].Other problems reported by the respondents included dizziness and headache, which were observed in two thirds of the seniors. These can have various backgrounds, from balance disturbances related to laryngological abnormalities to cardiovascular and respiratory system disorders. As aging affects the cardiovascular system significantly, atherosclerotic changes frequently occur in the elderly, leading to ischemia of the internal organs and various other disorders—particularly in the lower legs and feet, where hyperpigmentation, atrophy, and ulcerations often appear, and patients often feel cold, leading to seniors’ complaints of so-called heavy legs [22]; this was present in three quarters of the study population. However, the most vivid symptoms are those of coronary atherosclerosis, as the elderly demonstrate a tendency to fatigue easily, angina pectoris, breathlessness upon effort, and edema; as many as one third of the women in the study reported feeling tachycardia and chest pain. In elderly patients, atrophy of the alveolar wall occurs and dyspnea arises; this is an especially annoying and stressful symptom that lowers quality of life [15]; not surprisingly, this was included in the complaints of the study group.

Another problem reported by over 50% of the women in the research was back pain. The main reason for this may be the maintenance of a stable, unchanging body position and, in consequence, pressure being exerted on a certain body part. Moreover, the risk of back pain is aggravated by edema, inappropriate nutrition, excessively tight clothes, urinary and fecal incontinence, neglect of hygiene, diabetes, and disorders of the cardiovascular system [8]. A significant number of respondents also reported the occurrence of arthralgia, which can be a symptom of age-related degenerative changes in the motor system. The characteristic age-related changes include the weakening of muscle groups, degenerative phenomena within the spine, decreasing bone and muscle mass, and an increase in fat tissue [23, 24].

In the questionnaire we used, some questions assessed the condition of the nervous system, and determined whether depression symptoms were present in the research population. Depression symptoms are so common in the elderly that they seem to equate with the process of aging. In people aged over 65 years, depression can manifest as a deterioration in cognitive functions and, if extreme, may mimic dementia. In the initial stage, interest and pleasure in activities decrease, a feeling of disappointment is present, and suicidal thoughts are common [25]. In the study population, all of the above symptoms were present: one tenth reported suicidal thoughts. Depression symptoms in the elderly have many features in common with somatic diseases, which are very frequent at this age, and with pain, which often accompanies them. On the other hand, the majority of patients experience pain symptoms of unexplained etiology during depression episodes, which is not only the cause of many difficulties in taking histories for clinical and research purposes [26], but is also an important factor that significantly decreases the quality of life of the elderly [27, 28]. Although Hippocrates was the first to describe pain complaints in the course of depression, contemporary systems of disease classification—including the ICD-10 [29] and DSM-IV [30]—still do not enumerate pain as a depression symptom. However, as modern research has proved the simultaneity of these phenomena [31, 32], many pharmacological therapies used in patients with depression now often include an analgesic component [33, 34]. It has been proven that patients with depression report pain complaints considerably more frequently [35]. As this affect elderly people more frequently [36], it should be remembered that typical somatic age-related symptoms may point to depression. These symptoms—including a continual feeling of tiredness and a lack of energy, easily becoming fatigued, and appetite disturbances [37]—were mentioned by the women in the research group.

Another serious problem that can accompany depression is sleeplessness [37]; our results show that all individuals evaluated their sleep as not being of the highest quality; the best results were observed in the Polish group, followed by the German and the French groups. Many of the respondents complained of sleep disturbances, problems falling asleep, frequent waking during the night, and daytime sleepiness, which had a direct impact on the perception of pain and depressed mood. As the need for sleep in elderly people is decreased, physicians in nursing homes can use pharmacotherapy to improve residents’ comfort, but are not too quick to do so, as hypnotics can interact with other medication [8]. The French and German centers commonly implement a sleep diary, which records the number of sleepless hours a day. This method allows accurate verification of the level of sleepless and significantly reduces the amount of drugs administered.

Our research has shown that a significant number of the seniors did not present any symptoms of depression; the women living in France and Germany made up most of these. Unfortunately, symptoms of depression were found in a large group of respondents, most of whom suffered from them in mild forms. Symptoms of mild or medium-intense depression were most frequently observed among Polish residents, then among the German and the French. On the other hand, severe depression most often occurred among the German women, followed by those from Poland, and then France. Depression symptoms present a serious challenge for health care workers. Early diagnosis and proper treatment of depression brings good results and considerably improves the patients’ quality of life [10].

The DOLOPLUS scale included in the questionnaire allowed us to determine the type of pain occurring in the majority of nursing home residents. The results show that pain is a commonly observed phenomenon in people aged over 65 years, regardless of country of residence [38]. It is significant that most respondents complained of mild or medium pain, which may indicate that the analgesic therapy used was achieving its aim. The increased prevalence of pain among the elderly population may be related to the coexistence of many diseases [39]. A patient can simultaneously experience several types of pain: about four fifths of seniors feel at least two types of pain, while in a third there are at least four types. Pain cannot be considered only in physical terms, as it is a highly subjective and individual experience. A holistic approach demands that we view it as a psychosomatic phenomenon, which means that pain is what the patient describes, and not what health care institution workers understand by the notion. The feeling of pain is modified by numerous factors, several of which lower its threshold: these include fatigue, sleeplessness, unrest, anxiety, anger, sadness, and depression. However, other factors—including good sleep, a friendly environment, the presence of other people, and improvements in mood, as well as taking analgesic, anxiolytic, and antidepressant medication—make the threshold higher [19, 40]. Consequently, pain treatment should be individualized and introduced suitably early. Medication should be administered in the most efficient, least painful, and simplest way. Because of disturbances to the peripheral circulation, high levels of pain, and the risk of local changes, intramuscular application should be avoided in favor of subcutaneous injections in people aged over 65 years [22, 41]. This administration method is used at all the described centers, though in Poland less often than in France or Germany. Pain therapy was closely monitored in all three countries.

Study and assessment of the patient’s quality of life, as affected by pain and mood depression, are integral elements of assessing elderly patients, and constitute an important parameter of care efficiency. Improving the comfort of seniors is a challenge to everyone engaged in the care of the elderly, so assessment of quality of life is a helpful tool in selecting the most efficient therapeutic strategies, considering the interest of the patients themselves and the therapeutic care team. In all patients over 65 years of age, physical and mental health status should be assessed individually, as the majority present symptoms of various disease processes that constitute reasons for subjective physical complaints (pain) and psychological components (depression symptoms) [42, 43].

An objective assessment of quality of life is difficult and imprecise, because of the disabilities and comorbidities commonly present in this social group. For this reason, a subjective indirect index is often applied, allowing the health status to be determined by the patients themselves; this proves to be a reliable tool [42]. Most of the surveyed seniors rated their health as low; the lowest assessments were reported among the French, then among the German, and then among the Polish women—most probably because the respondents in the Polish centers were the youngest. Additionally, the problems described by the seniors contribute to difficulties in everyday functioning: most of the Polish women, being the youngest, pointed to smaller difficulties, while the French and German women depicted their everyday functioning as very burdensome. In interpreting these differences, however, one should also consider information on the seniors’ varied expectations.

In summary, it can be stated that there are differences in the detailed quality of life assessment provided by elderly women residing in institutions in different European countries. Nevertheless, the differences are not significant and the core values are comparable. Pain—so important in seniors’ quality of life—is common to all respondents [44]. It constitutes a serious and widespread problem, whose solution might be facilitated through international cooperation in the realm of specific care for seniors. The experience of countries with more extensive practice in providing comprehensive care for the elderly can be extremely helpful in shaping modern institutional care in other regions. Although taking care of the physical problems of the elderly is important, their psychological condition should also be considered, as it is responsible for their ability to monitor the situation, as well as their thoughts and behaviors. Emotional experience, general feeling, and the ability to function in everyday life all have significant effects on seniors’ general condition and their quality of life experience.

## Conclusions

There are no significant differences between the prevalence of pain and symptoms of depression in elderly people living in institutions in the examined European countries; this also refers to the average quality of life of the residents.

The decrease in the quality of life is mainly due to various complaints and pain. There is a close relationship between health status and quality of life.

Further research should be performed on the interdependencies between the occurrence of pain and depression, including the primary reasons that lead to both phenomena. The recognition of factors that induce pain complaints and mood depression in elderly people will contribute to improving their comfort.

## Data Availability

All the data generated and examined in study are included in this published article. The datasets used and/or analyzed during the current study are available from the corresponding author on reasonable request.

## Abbreviations

BDI: Beck depression inventory
DOLOPLUS: Scale of behavioral pain assessment in the elderly
SD: Standard deviation
